# Effects of supplemental xylanase and xylooligosaccharides on production performance and gut health variables of broiler chickens

**DOI:** 10.1186/s40104-021-00617-8

**Published:** 2021-09-06

**Authors:** Amit K. Singh, Birendra Mishra, Michael R. Bedford, Rajesh Jha

**Affiliations:** 1 Department of Human Nutrition, Food and Animal Sciences, University of Hawaii at Manoa, Honolulu, HI 96822 USA; 2 AB Vista Feed Ingredients, Marlborough, Wiltshire UK

**Keywords:** Gene expression, Histomorphology, Microbiota, Poultry, Prebiotics, Short-chain fatty acid, Xylooligosaccharide

## Abstract

**Background:**

This study evaluated the effects of supplemental xylanase and xylooligosaccharides (XOS) in a corn-soybean meal (SBM)-based diet on growth performance and intestinal health of broilers. A total of 288 day-old chicks (Cobb 500) were allocated to 36 floor pens (8 birds/pen) equally in 9 dietary treatments in a 3 × 3 factorial arrangement. The treatments were combinations of 3 levels of xylanase (0, 0.005% and 0.01% Econase XT) and 3 levels of prebiotics (0, 0.005% and 0.01% XOS) added to basal mash diets formulated in three phases (starter, d 0–14; grower, d 15–28; finisher, d 29–42). The feed intake and body weights were recorded weekly. On d 42, ileal sections were collected for histomorphometric and gene expression analysis, and cecal content was collected for determining short-chain fatty acids (SCFA) and microbiota.

**Results:**

Xylanase linearly (*P* < 0.01) increased the average daily gain (ADG) in both the finisher and total period and the final body weight gain (FBWG, 2940 & 2932 vs. 2760 g) of broilers. XOS did not significantly increase either ADG or FBWG (*P* > 0.05). Supplemental xylanase and XOS did not affect average daily feed intake and feed conversion ratio (*P* > 0.05). Xylanase and XOS did not change villus height (VH) or crypt depth (CD) ratio (*P* > 0.05). However, xylanase exhibited a trend (*P* = 0.097) on VH:CD ratio. The inclusion of 0.01% XOS without xylanase increased the level of IL-10 (a marker of anti-inflammatory cytokine) and IL-4 (a T-cell differentiation cytokine) genes compared with control (*P* < 0.05). The acetate production was increased by xylanase (*P* < 0.01) and XOS (*P* < 0.05) without an additive effect. Xylanase increased total SCFA (*P* < 0.01) while XOS had a tendency to increase (*P* = 0.052). Alpha and beta diversity of microbiota among treatments were not different (*P* > 0.05). However, the mean proportion of family *Ruminococcaceae* was increased by the supplemental 0.01% xylanase (*P* < 0.01).

**Conclusion:**

It can be concluded that XOS can enhance cecal fermentation, while xylanase can increase the body weight gain along with the fermentation metabolites in the ceca of broilers fed the corn-SBM-based diet but the effects may not always translate into an improved mucosal absorptive capacity and a better feed efficiency.

**Supplementary Information:**

The online version contains supplementary material available at 10.1186/s40104-021-00617-8.

## Introduction

The application of exogenous enzymes in fibrous diets has been found to increase nutrient utilization and subsequently improve the growth performance of broilers [1, 2]. However, the response of different enzymes is not consistent, and it could vary based on the types of ingredients present in the diet. Among the exogenous enzymes, xylanase is commonly used to improve non-starch polysaccharide (NSP) degradation in wheat-based diets, but it is also increasingly being used in corn-based diets in broilers [3, 4]. The enhancement in digestibility and the improvement in nutrient utilization are essential for the improved growth performance. For better nutrient utilization, proper functioning of the immune system and overall good gut health are essential in broilers. To ensure improved nutrient utilization, it requires an increased mucosal surface area for absorption, a better access of digestive enzymes to nutrients in the digesta, and increased NSP degradation in the host [5]. Exogenous enzymes like xylanase could reduce the intestinal digesta viscosity, enhance the absorption of nutrients and concurrently increase the rapidly fermentable substrates for the normal gut microbiota [6].

Feeding xylanase increases the contents of xylooligosaccharides (XOS) and xylose in the colon and ceca through the hydrolysis of polymeric xylans and influences the cecal microbiome to increase the fermentation metabolites [7–9]. The short-chain oligosaccharides provide substrates to establish the commensal microbiome in the lower gut [10, 11]. Xylanase has been reported to increase *Lactobacillus* spp. counts in the intestine, reduce gram-positive cocci and enterobacteria, and increase the production of butyrate in broilers [12]. In addition to the improvement by xylanase, prebiotics can also influence the health and growth performance of broilers by modulating and supporting the growth of beneficial intestinal microbiota [13]. Prebiotics can stimulate immunity either directly or through the fermentation metabolites produced in response to the enhanced colonization of GIT by beneficial microbiota [5]. Besides other oligosaccharides, XOS is being studied as a potential prebiotic since it had been shown to promote the production of butyrate by cross-feeding of lactate to butyrate-producing bacteria and support the intestinal mucosa of broilers [14]. Moreover, XOS supplementation increases short-chain fatty acids (SCFA), stimulates the immune system, and increases the population of beneficial bacteria in chickens [15, 16]. Increased production of fermentation metabolites like SCFA can enhance the growth of intestinal villi and improve mucosal health. Besides supporting the gut microbiome, XOS is also reported to enhance the immune response by maintaining antibody titers, boost endocrine metabolism, and improve growth performance [17].

However, further studies are necessary to ascertain the roles of xylanase and XOS in improving immune response, modulating microbiota in the ceca, and enhancing fermentation metabolites in broilers. Moreover, to supplement xylanase and XOS in regular feed production, a thorough evaluation of their interaction effect on the gut health and growth performance of broilers is required for determining their optimum inclusion level. The objective of this study was to investigate the effects of supplementing various levels of xylanase and XOS on growth performance, ileal histomorphology, immune response, cecal fermentation metabolites, and cecal microbiome of broilers.

## Materials and methods

### Experimental design and dietary treatments

A growth performance study in floor pens was conducted at the Small Animal Facility of the University of Hawaii at Manoa. This study was carried out following the approved protocol from the Institutional Animal Care and Use Committee of the University of Hawaii. The diets were formulated with the same basal composition using corn and soybean meal in three phases: starter for d 0–14, grower for d 15–28, and finisher for d 29–42 (Table 1). The nutrient composition of the diets was managed to meet the requirements of broilers [18]. The formulated diets had 3 levels of xylanase enzyme (Econase XT 0 g/t—0 or no enzyme, Econase XT 50 g/t—0.005% or 8,000 BXU/kg Econase XT, and Econase XT 100 g/t—0.01% or 16,000 BXU/kg Econase XT) and 3 levels of XOS prebiotic (XOS 0 g/t or 0%, XOS 50 g/t or 0.005%, and XOS 100 g/ t or 0.01%) supplemented in a completely randomized design in 3 × 3 factorial arrangement. Thus, the combination of xylanase and XOS produced 9 dietary treatments. Phytase (500 FTU/kg of Quantum blue; AB Vista Feed Ingredients, Marlborough, Wiltshire, UK), with a matrix value of 0.16% Ca and 0.15% P, was added in all diets. The diets were prepared and fed as mash to the birds in all treatment groups.

**Table 1:** The composition of ingredients and nutrient content of diets supplemented with xylanase and xylooligosachharides and fed in different phases to broiler from d 0-42 (as-fed basis, g/kg unless otherwise indicated)

	Inclusion level^b^		
	Starter	Grower	Finisher
	(d 0-14)	(d 15-28)	(d 29-42)
**Ingredients, g/kg**			
Corn	572.70	613.70	641.50
SBM-48	360.00	320.00	290.00
Soybean oil	28.00	31.00	37.00
Limestone	14.00	14.00	13.00
Monocalcium phosphate	9.00	6.50	5.00
Lysine	3.50	3.00	2.50
Methionine	2.50	1.90	1.40
Threonine	1.00	1.00	1.00
Nacl	3.00	2.80	2.50
Sodium bicarbonate	1.20	1.00	1.00
Vitamin+mineral mix^a^	5.00	5.00	5.00
Phytase	0.10	0.10	0.10
**Calculated content, g/kg**			
ME, MJ/kg	12.83	13.11	13.43
Crude protein	222.80	204.77	190.85
NDF	79.20	79.61	79.65
Crude fat	26.64	25.98	25.42
dig Lys	13.50	12.13	11.00
digMet	5.61	4.84	4.22
dig Thr	7.54	7.01	6.61
Trp	3.01	2.74	2.53
Meth+Cysteine	10.20	9.26	8.48
Arg	15.28	14.08	13.17
Val	10.86	10.17	9.65
Ile	9.29	8.56	8.01
Leu	19.19	18.11	17.26
Ca	9.40	8.85	8.12
Total P	7.23	6.57	6.15
Available P	4.55	3.99	3.65
Na	2.01	1.87	1.75
Cl	2.22	2.09	1.91
Choline	1.34	1.25	1.19
**Analyzed composition, as is, g/kg**			
DM	877.6	876.2	882.0
Gross energy, MJ/kg	15.86	16.11	16.4
Crude protein	227.82	210.91	192.95
Crude fat	31.10	37.77	52.78
Crude fiber	22.93	22.44	22.00
ADF	34.46	33.09	39.60
NDF	77.25	78.13	85.97
Ash	53.14	48.27	46.82

^a^Provides following nutrients (per kg of diet): vitamin A (trans-retinyl acetate), 10,000 IU; vitamin D_3_ (cholecalciferol), 3,000 IU; vitamin E (all-*rac*-tocopherol-acetate), 30 mg; vitamin B_1_, 2 mg; vitamin B_2_, 8 mg; vitamin B_6_, 4 mg; vitamin B_12_ (cyanocobalamin), 0.025 mg; vitamin K_3_ (bisulphatemenadione complex), 3mg; choline (choline chloride), 250 mg; nicotinic acid, 60 mg; pantothenic acid (D-calcium pantothenate), 15 mg; folic acid, 1.5 mg; betaíne anhydrous, 80 mg; D-biotin, 0.15 mg; zinc (ZnO), 80 mg; manganese (MnO), 70 mg iron (FeCO_3_), 60 mg; copper (CuSO_4_·5H_2_O), 8 mg; iodine (KI), 2 mg; selenium (Na_2_SeO_3_), 0.2 mg.

^b^The basal diets in all phases were top-dressed with the combination of 3 levels of xylanase (0 BXU/kg, 8,000 BXU/kg and 16,000 BXU/kg) and 3 levels of xylooligosaccharides (0 g/t, 50 g/t and 100 g/t) to yield 9 treatments

### Experimental methods and housing arrangement

A total of 288 one-day-old unsexed Cobb 500 broiler chicks were used. All birds were raised in standard broiler rearing environments in floor pens. The chicks were randomly allocated to 36 pens so that each pen had 8 birds, and 4 pens were assigned to each treatment. The feed and water were provided ad libitum to the birds in all pens. The feed intake and the weight of birds were measured weekly from d 0 till d 42. The average daily gain (ADG), average daily feed intake (ADFI), and feed conversion ratio (FCR) were calculated from the data for all three phases and total period. The daily mortality was recorded along with feed intake to adjust the FCR for each pen. On d 42, the body weight of the birds was recorded, and 2 birds from each pen, making 8 birds for each treatment, were euthanized using CO_2_. The cecal contents were collected, snap-frozen, and stored at − 20 °C until further analysis for SCFA. Simultaneously, another set of cecal contents were collected from 1 bird per pen in cryovials and snap-frozen and transferred to a − 80 °C freezer for later DNA extraction and microbiota analysis. Next, approximately 2–3 cm section of the ileum (defined as the section between Meckel’s diverticulum and ileocecal junction) was cut from 2 birds and fixed in 10% buffered formalin for histomorphometry. Moreover, the weight of the liver, gizzard, proventriculus, drumsticks, whole breast, and abdominal fat was recorded from 1 bird per pen for each treatment to calculate the relative organ and carcass weight (% of live body weight).

### Proximate analysis and enzyme activity

The nutrient profile of feed samples was analyzed for dry matter (DM), gross energy (GE), N (for crude protein, CP), crude fat, ash, crude fiber, neutral detergent fiber (NDF), and acid detergent fiber (ADF) using the standard procedures of Association of Official Agricultural Chemists [19] (Table 1). The enzyme activity levels in the final feed samples (200 g) were measured at the AB Vista Innovation and Technology Center (Tredomen Park, Ystrad Mynach, UK) in duplicate and were reported as activity units. The amount of enzyme that produces 1 nmol of xylan, a reducing sugar from birch xylan in 1 s at 50 °C at pH 5.3 is defined as one xylanase units (BXU). The enzyme activity recovery in the final feed of all phases in the no xylanase groups was less than 2,000 BXU/kg. Xylanase activity was about 100%–115% of expected levels in the starter, about 95%–110% in grower, and about 95%–105% in the finisher diets, thus confirmed the activity to be within 15% of the targeted levels.

### Chromatographic analysis of short-chain fatty acids

The cecal digesta was analyzed for major SCFA by gas chromatography using GC system (Trace 1300, Thermo Scientific, Waltham, MA, USA) equipped with a flame ionization detector and AS 1310 series automatic liquid sampler, according to Singh et al. [4]. A calibration curve of the external standard was made in the range of 0–8 mmol/L using SCFA mix (Sigma-Aldrich, St. Louis, MO, USA). Trimethylacetic acid (Sigma-Aldrich, St. Louis, MO, USA) was used as an internal standard. The standard SCFA mix was analyzed by GC to get an individual calibration curve for each external standard compound based on their response ratio to the internal standard. SCFA data handling and processing were performed on ChromeleonTM 7.2 software (Thermo Scientific, Waltham, MA, USA).

### Histomorphometry of ileal mucosa

The formalin-fixed ileal samples (*n *= 4 per treatment) were transferred to 70% ethanol the next day, and embedding, sectioning (5 µm) and Hematoxylin and Eosin (H&E) staining was done at John A. Burns School of Medicine histology core of University of Hawaii at Manoa. Each slide had 6 ileal cross-sections, and 3 well-oriented sets of villus and crypts for each section was observed at 8× magnification of objective under a microscope (Olympus BX43, Olympus Co, Tokyo, Japan) fitted with camera and image processing Infinity Analyze software (Lumenera Corporation, Ottawa, ON, Canada). The villus height (VH) was measured from the tip of mucosal projection to the invagination creating a valley between two mucosal projections. The crypt depth (CD) was measured from the opening of the invagination to the base above the lamina muscularis mucosae. The VH:CD ratio was calculated, and the average of each pen was recorded.

### RNA extraction and gene expression analysis

Frozen ileal tissue (50–100 mg) was homogenized in 300 µl of TRIzol® (Invitrogen, Carlsbad, CA) with 9–11 Zirconia beads (BioSpec Products, Bartlesville, OK, USA) of 2.3 mm diameter. The homogenization was carried out by bead beating in a nuclease-free safe lock Eppendorf tube on a vertical Bullet Blender® (Next Advance, Inc. Troy, NY) for 2–3 min at speed 8. Next, the homogenized sample was centrifuged at 10,000 r/min at 4 °C for 1 min, and 250 µL of the supernatant was pipetted and mixed with 750 µL of TRIzol® in a new microcentrifuge tube. Total RNA extraction, quality assessment, and cDNA preparation were done according to the procedure described by Wasti et al. [20]. The primers used in this study were designed on the NCBI primer blast tool for immune gene markers that were used previously [21]. The program for amplification was set as 50 °C for 2 min then 95 °C for 2 min that was followed by 40 cycles at 95 °C for 15 s for denaturation, annealing at 60 °C for 15 s, and extension at 72 °C for 1 min. After 40 cycles of qPCR, melt curves were generated to ensure the specificity of the primers used. The target genes were analyzed in duplicate, and the Beta-actin (β-actin) housekeeping gene was analyzed in triplicate, and an average value was recorded for all experimental replicates. After the qPCR run was complete, the cycle of threshold (Ct) was noted for each gene marker. The gene expression was calculated as relative quantification for each target gene using the base 2 exponential negative delta- delta Ct (2^−ΔΔCt^) method. The mean ΔCt of 0% xylanase + 0% XOS was used to calculate the ΔΔCt of each treatment.

### Bioinformatic analysis of 16S rRNA amplicon

Cecal microbial DNA was extracted using QIAamp® Fast DNA Stool Mini Kit (Qiagen, Hilden, Germany) according to the manufacturer’s instructions. The library preparation was performed by amplicon PCR with primers targeting V3–V4 variable regions of the 16S rRNA gene. The forward primer sequence was 5′-TCGTCGGCAGCGTCAGATGTGTATAAGAGACAGCCTACGGGNGGCWGCAG-3′ and reverse primer sequence was 5′-TCTCGTGGGCTCGGAGATGTGTATAAGAGACAGGACTACHVGGGTATCTAATCC-3′, containing Illumina overhang adapter and locus-specific sequence for 16S rRNA amplification [22]. The amplicon PCR, product cleaning, and attaching of Nextera XT dual indices to the amplicon was performed as previously described by Singh et al. [4].

For further processing, Quantitative Insights Into Microbial Ecology (QIIME™ version 2.0 release 2019.4) was used to import demultiplexed paired-end reads of 300 bp in length for all samples [23]. After importing into QIIME2, the DADA2 pipeline was used to denoise, trim, and filter these paired-end sequences. The filtered sequences were subjected to align-to-tree-mafft-fasttree pipeline from the QIIME phylogeny plugin to generate an unrooted and rooted tree for phylogeny. A Naïve Bayes classifier pre-trained on the Greengenes 13_8 99% OTU was used for taxonomy analysis. The diversity plugin method named core-metrics-phylogenetic was used to conduct alpha and beta diversity analysis on sampling depth of 10,000 frequency. Alpha diversity results were presented as Shannon Index and observed OTUs, while Bray Curtis metrics and unweighted UniFrac were applied for beta diversity. Moreover, we accessed the Clusters of Orthologous Groups of proteins (COGs) and the Kyoto Encyclopedia of Genes and Genomes (KEGG; Uji, Kyoto, Japan) databases to conduct a Phylogenetic Investigation of Communities by Reconstruction of Unobserved States (PICRUSt) to determine the effect of xylanase and XOS on the predictive functional profile of cecal microbiota. For this, a closed-reference OTU table was generated based on the greengenes database. Once the biome file was obtained, a software package Statistical Analysis of Taxonomic and Functional Profiles version 2.1.3 was employed for the processing and presentation of the mean differences [24].

### Statistical analysis

All the data for growth performance variables, relative carcass and organ weight, ileal histomorphometry, and SCFA were subjected to the MIXED procedure of SAS V9.2 (SAS Institute Inc., Cary, NC, USA) to compare the effects of treatments. A probability of *P* < 0.05 was considered significant for differences among treatments means that was further separated by the Tukey test using pdmix macro of SAS. The log-transformation was performed for all the fold change data of immune gene expression, and TTest procedure of SAS was applied to compare test variables with control variables. For analysis of microbiota diversity, Kruskal–Wallis pairwise test for alpha diversity and pairwise PERMANOVA for beta diversity were implemented in QIIME 2. White’s non-parametric t-test was run for statistical analysis of the predicted functional pathways [25]. The correlogram showing association among various biological parameters was generated using corrplot package, while the heatmap was constructed using pheatmap package of R software based on Spearman’s rank correlation. The level of significance for biological parameters and microbiota association was set at *P* < 0.05.

## Results

The calculated nutrients in the feed formulation in different phases diet were similar to the proximate estimates (Table 1). Xylanase and XOS had no interaction effects on growth parameters and did not vary ADFI and FCR from d 0–42 in any rearing phases (*P* > 0.05; Table 2). However, during the total period, a trend (*P* = 0.081) was observed for the effect of xylanase on ADFI, where the increasing level of xylanase increased the ADFI. In the finisher and total period, ADG was linearly (*P* < 0.01) increased by xylanase. Xylanase also linearly (*P* < 0.01) increased the final body weight gain (FBWG) of broilers by more than 170 g (2940 & 2932 vs. 2760 g) compared with the control at d 42 (*P* < 0.05). In contrast, the numerically higher ADG and FBWG in the XOS groups were not statistically different from the control (*P* > 0.05). The effects of xylanase, XOS, and their interaction were not significantly different (*P* > 0.05) for the relative weight of the liver, gizzard, proventriculus, drumsticks, breast, and abdominal fat (Table 3). Ileal mucosa VH and CD were not affected by treatments (*P* > 0.05; Table 4). Xylanase exhibited a trend on the VH to CD ratio (*P* = 0.097). On d 42, xylanase and XOS increased (*P* < 0.05) the concentration of acetate in the ceca in a dose-dependent manner, but no interaction was observed for any SCFA (Table 5). Xylanase and XOS did not affect the cecal concentration of butyrate and propionate (*P* > 0.05). While XOS had a trend (*P* = 0.052), xylanase significantly (*P* < 0.01) increased the cecal concentration of the total SCFA (*P* < 0.01).

**Table 2:** Effects of supplemental xylanase and xylooligosaccharides on growth performance of broilers in different dietary phases from d 0–42 post-hatch

Treatments	Variables												
	ADFI, g/day				ADG, g/day				FCR				FBWG, g
	0–14	15–28	29–42	0–42	0–14	15–28	29–42	0–42	0–14	15–28	29–42	0–42	0–42
0 BXU xylanase													
Xylo-oligo, 0 g/t	36.0	109.0	171.2	105.4	27.0	75.6	91.9	64.9	1.34	1.44	1.87	1.63	2,694
Xylo-oligo, 50 g/t	36.3	109.0	175.7	107.0	27.9	76.7	94.7	66.4	1.31	1.42	1.86	1.61	2,800
Xylo-oligo, 100 g/t	36.4	109.9	173.7	106.7	28.2	77.5	92.9	66.2	1.29	1.42	1.88	1.61	2,786
8,000 BXU xylanase													
Xylo-oligo, 0 g/t	36.3	110.5	177.5	108.1	27.7	77.5	97.9	67.7	1.31	1.43	1.81	1.60	2,890
Xylo-oligo, 50 g/t	36.6	110.9	179.3	108.9	28.3	79.7	96.9	68.3	1.30	1.39	1.86	1.60	2,959
Xylo-oligo, 100 g/t	36.7	111.6	179.2	109.2	28.7	80.2	98.6	69.2	1.28	1.39	1.82	1.58	2,972
16,000 BXU xylanase													
Xylo-oligo, 0 g/t	36.7	111.4	179.4	109.2	28.5	78.3	98.8	68.5	1.29	1.43	1.82	1.60	2,909
Xylo-oligo, 50 g/t	36.7	112.2	178.4	109.1	28.8	79.9	98.9	69.2	1.28	1.41	1.82	1.58	2,946
Xylo-oligo, 100 g/t	36.8	111.2	179.2	109.0	29.1	80.5	98.5	69.4	1.26	1.38	1.82	1.57	2,941
SEM (*n* = 4)	0.50	1.70	3.47	1.56	0.87	1.76	2.44	1.33	0.04	0.02	0.04	0.02	65.02
Main effect factors													
Xylanase													
0 BXU xylanase	36.2	109.3	173.5	106.4	27.7	76.6	93.2^b^	65.8^b^	1.31	1.43	1.87	1.62	2,760^b^
8000 BXU xylanase	36.5	111.0	178.7	108.7	28.3	79.1	97.8^ab^	68.4^ab^	1.30	1.40	1.83	1.59	2,940^a^
16,000 BXU xylanase	36.7	111.6	179.0	109.1	28.8	79.6	98.7^a^	69.0^a^	1.28	1.40	1.82	1.58	2,932^a^
Xylooligosaccharides													
Xylo-oligo, 0 g/t	36.3	110.3	176.0	107.6	27.7	77.1	96.2	67.0	1.31	1.43	1.84	1.61	2,831
Xylo-oligo, 50 g/t	36.5	110.7	177.8	108.3	28.3	78.8	96.8	68.0	1.29	1.41	1.84	1.59	2,902
Xylo-oligo, 100 g/t	36.6	110.9	177.4	108.3	28.7	79.4	96.7	68.3	1.28	1.40	1.84	1.59	2,900
SEM (*n* = 12)	0.29	0.98	2.01	0.90	0.50	1.02	1.41	0.77	0.02	0.01	0.02	0.01	37.54
*P*-value													
Xylanase	0.490	0.247	0.114	0.081	0.317	0.108	0.020	0.015	0.480	0.395	0.305	0.197	0.003
Linear	–	–	–	–	–	–	0.009	0.006	–	–	–	–	0.003
Quadratic	–	–	–	–	–	–	0.290	0.317	–	–	–	–	0.050
Xylo-oligo	0.748	0.922	0.809	0.790	0.417	0.280	0.948	0.500	0.524	0.244	0.973	0.606	0.335
Xylanase × Xylo-oligo	0.993	0.978	0.959	0.985	0.998	0.999	0.935	0.992	0.990	0.980	0.954	0.995	0.983

0 BXU xylanase = 0 g xylanase/ t feed, 8,000 BXU xylanase = 50 g xylanase/ t feed, 16,000 BXU xylanase = 100 g xylanase/ t feed. Xylo-oligo: xylooligosaccharides. *ADFI*: average daily feed intake, *ADG*: average daily gain, *FCR*: feed conversion ratio, *FBWG*: final body weight gain

^a-b^Within columns in the xylanase main effect, means without a common superscript differ (*P* < 0.05)

**Table 3:** Effects of xylanase and xylooligosaccharides inclusion on relative organ weight (g/100 g live weight) of broilers from d 0–42 post-hatch

	Variables					
	Liver	Proventriculus	Gizzard	Breast	Drumstick	Abdominal fat
Treatments						
0 BXU Xylanase						
Xylo-oligo, 0 g/t	2.10	0.30	1.35	30.01	4.83	0.79
Xylo-oligo, 50 g/t	2.02	0.32	1.34	29.06	4.36	1.34
Xylo-oligo, 100 g/t	2.21	0.28	1.24	29.68	4.78	1.48
8000 BXU Xylanase						
Xylo-oligo, 0 g/t	2.20	0.31	1.18	30.03	4.58	1.27
Xylo-oligo, 50 g/t	2.23	0.29	1.20	29.85	4.46	1.09
Xylo-oligo, 100 g/t	2.07	0.27	1.11	29.98	4.22	1.08
16,000 BXU Xylanase						
Xylo-oligo, 0 g/t	2.53	0.26	1.09	29.01	4.50	1.31
Xylo-oligo, 50 g/t	2.22	0.27	1.08	30.90	4.70	1.21
Xylo-oligo, 100 g/t	2.11	0.30	1.40	27.81	4.70	1.55
SEM (*n* = 4)	0.213	0.019	0.094	1.079	0.162	0.188
Main effect factors						
Xylanase						
0 BXU Xylanase	2.11	0.30	1.31	29.58	4.66	1.20
8000 BXU Xylanase	2.17	0.29	1.16	29.95	4.42	1.15
16,000 BXU Xylanase	2.29	0.28	1.19	29.24	4.63	1.36
Xylo-oligo						
Xylo-oligo, 0 g/t	2.28	0.29	1.20	29.68	4.64	1.12
Xylo-oligo, 50 g/t	2.16	0.29	1.21	29.94	4.51	1.21
Xylo-oligo, 100 g/t	2.13	0.28	1.25	29.16	4.57	1.37
SEM (*n* = 12)	0.123	0.011	0.054	0.623	0.094	0.108
*P*-value						
Xylanase	0.595	0.298	0.155	0.723	0.162	0.378
Xylo-oligo	0.673	0.899	0.808	0.669	0.622	0.279
Xylanase × Xylo-oligo	0.740	0.302	0.102	0.452	0.135	0.156

0 BXU xylanase = 0 g xylanase/ t feed, 8,000 BXU xylanase = 50 g xylanase/ t feed, 16,000 BXU xylanase = 100 g xylanase/ t feed. Xylo-oligo: xylooligosaccharides

**Table 4:** Effects of supplemental xylanase and xylooligosaccharides on ileum histomorphology of broiler chickens on d 42 post-hatch

Treatments	Variables, µm		
	VH	CD	VH: CD
0 BXU xylanase			
Xylo-oligo, 0 g/t	865.2	98.7	8.9
Xylo-oligo, 50 g/t	817.5	87.8	9.4
Xylo-oligo, 100 g/t	879.7	92.7	9.6
8000 BXU xylanase			
Xylo-oligo, 0 g/t	883.9	96.4	9.3
Xylo-oligo, 50 g/t	995.4	102.4	9.8
Xylo-oligo, 100 g/t	886.0	91.6	9.8
16,000 BXU xylanase			
Xylo-oligo, 0 g/t	963.4	98.2	9.9
Xylo-oligo, 50 g/t	1,101.6	111.3	10.0
Xylo-oligo, 100 g/t	928.8	93.0	10.1
SEM (*n* = 4)	82.30	7.31	0.38
Main effect factors			
Xylanase			
0 BXU xylanase	854.1	93.0	9.3
8000 BXU xylanase	921.8	96.8	9.6
16,000 BXU xylanase	997.9	100.8	10.0
Xylooligosaccharides			
Xylo-oligo, 0 g/t	904.2	97.7	9.3
Xylo-oligo, 50 g/t	971.5	100.5	9.7
Xylo-oligo, 100 g/t	898.2	92.4	9.8
SEM (*n* = 12)	47.51	4.22	0.22
*P*-value			
Xylanase	0.120	0.438	0.097
Xylo-oligo	0.489	0.399	0.248
Xylanase × Xylo-oligo	0.646	0.468	0.965

VH: villus height, CD: crypt depth, Xylo-oligo: xylooligosaccharides, µm: micrometer

0 BXU xylanase = 0 g xylanase/ t feed, 8,000 BXU xylanase = 50 g xylanase/ t feed, 16,000 BXU xylanase = 100 g xylanase/ t feed

**Table 5:** Effects of supplemental xylanase and xylooligosaccharides on the production of cecal short-chain fatty acids (mmol/kg wet digesta) in broilers on d 42 post-hatch

Treatments	Variables, mmol/kg			
	Acetate	Propionate	Butyrate	Total SCFA
0 BXU xylanase				
Xylo-oligo, 0 g/t	50.2	5.4	6.3	64.9
Xylo-oligo, 50 g/t	58.5	4.7	10.0	76.2
Xylo-oligo, 100 g/t	57.2	5.5	10.8	76.8
8000 BXU xylanase				
Xylo-oligo, 0 g/t	68.0	6.5	9.7	87.5
Xylo-oligo, 50 g/t	66.6	5.9	8.0	84.1
Xylo-oligo, 100 g/t	71.0	6.0	10.1	90.7
16,000 BXU xylanase				
Xylo-oligo, 0 g/t	70.9	5.8	9.7	89.8
Xylo-oligo, 50 g/t	77.0	6.3	11.0	97.8
Xylo-oligo, 100 g/t	80.4	5.3	9.3	98.4
SEM (*n* = 4)	2.74	0.92	1.18	3.82
Main effect factors				
Xylanase				
0 BXU xylanase	55.3^c^	5.2	9.0	72.6^c^
8000 BXU xylanase	68.5^b^	6.1	9.3	87.4^b^
16,000 BXU xylanase	76.1^a^	5.8	10.0	95.3^a^
Xylooligosaccharides				
Xylo-oligo, 0 g/t	63.0^b^	5.9	8.5	80.7^b^
Xylo-oligo, 50 g/t	67.4^ab^	5.6	9.7	86.0^ab^
Xylo-oligo, 100 g/t	69.6^a^	5.6	10.1	88.6^a^
SEM (*n* = 12)	1.58	0.53	0.68	2.21
*P*-value				
Xylanase	< 0.001	0.479	0.558	< 0.001
Xylo-oligo	0.022	0.923	0.264	0.052
Xylanase × Xylo-oligo	0.409	0.907	0.102	0.404

0 BXU xylanase = 0 g xylanase/ ton feed, 8,000 BXU xylanase = 50 g xylanase/ ton feed, 16,000 BXU xylanase = 100 g xylanase/ ton feed

Xylo-oligo: xylooligosaccharides, SCFA: short chain fatty acid

^a-c^Within columns in the xylanase and xylooligosaccharides main effects, means without a common superscript differ (*P* < 0.05)

**Fig. 1:**
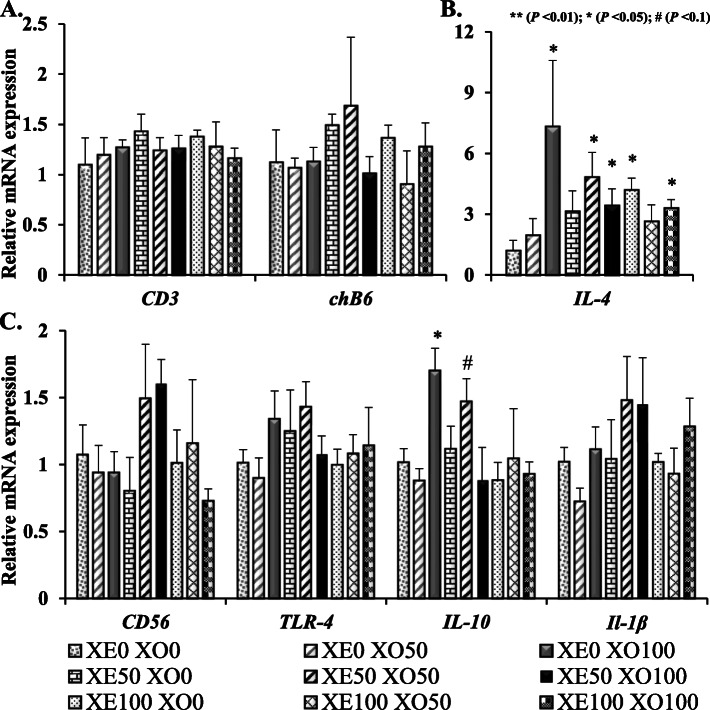
Effects of xylanase (XE) and xylooligosaccharides (XO) inclusion in feed on ileal gene markers of immune cells of broilers at d 42 post-hatch. The expression of each gene was examined using RT-qPCR and expressed as fold change ratio to the β-actin housekeeping gene, with the level being set to 1 in broilers from xylanase 0 + xylooligosaccharides 0 group. XE0 = xylanase 0 BXU/kg or 0 g per ton feed, XE50 = xylanase 8,000 BXU/kg or 50 g per ton feed, XE100 = xylanase16,000 BXU/kg or 100 g per ton feed. XO0 = 0 g xylooligosaccharides per ton feed, XO50 = 50 g xylooligosaccharides per ton feed, XO100 = 100 g xylooligosaccharides per ton feed. *CD3* (cluster of differentiation 3) is a marker gene for T-cell. *chB6* is a B-cell marker gene. IL-4 is a T-cell differentiation cytokine. *CD56* (known as neural cell adhesion molecule) is a gene marker for natural killer cells. *TLR-4* (Toll-like receptor 4) is a gene marker for macrophages. IL-10 (interleukin 10) is an anti-inflammatory cytokine. IL-1β is a pro-inflammatory cytokine

**Fig. 2:**
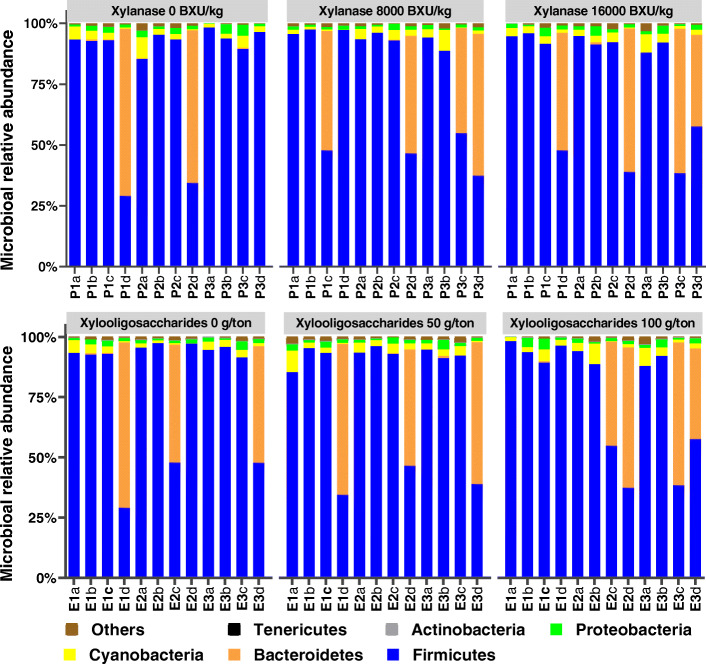
Stacked bar graph displays a comparison of microbial relative abundance (%) at phylum level among main effects (xylanase and xylooligosaccharides) of treatments in broilers at d 42 post-hatch. The label P denotes xylooligosaccharides prebiotics, and the label E denotes xylanase enzyme for each sample. The letters (a, b, c, and d) denote replicate samples. P1: 0 g xylooligosaccharides per ton feed, P2: 50 g xylooligosaccharides per ton feed, P3: 100 g xylooligosaccharides per ton feed. E1: xylanase 0 BXU/kg or 0 g per ton feed, E2: xylanase 8,000 BXU/kg or 50 g per ton feed, E3: xylanase16,000 BXU/kg or 100 g per ton feed

**Fig. 3:**
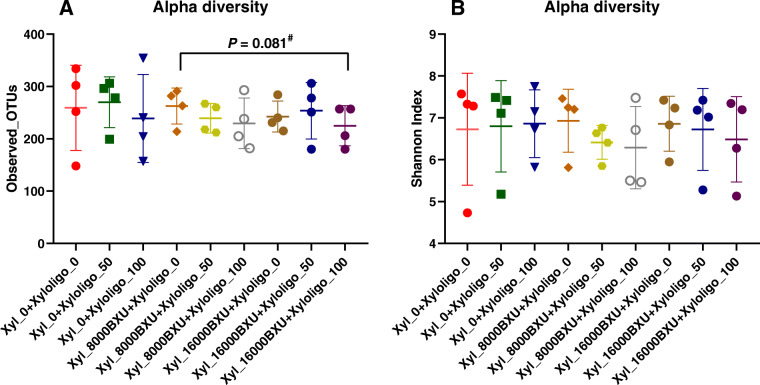
Alpha diversity analysis of different treatments at 10,000 reads depth per sample of cecal contents of broilers from d 42 post-hatch. **a** Observed OTUs **b** Shannon Index. Data represent mean ± SE. Statistical analyses were performed using Kruskal–Wallis test. Only the lowest p-value is shown. Xyl, xylanase; Xyloligo, xylooligosaccharides. Xyl_0: xylanase 0 BXU/kg feed, xyl_8,000BXU: xylanase 8,000 BXU/kg feed, xyl_16,000BXU: xylanase 16,000 BXU/kg feed, xylooligo_0: xylooligosacharides 0 g/t feed, xylooligo_50: xylooligosaccharides 50 g/t feed, and xylooligo_100: xylooligosaccharides 100 g/t feed

**Fig. 4:**
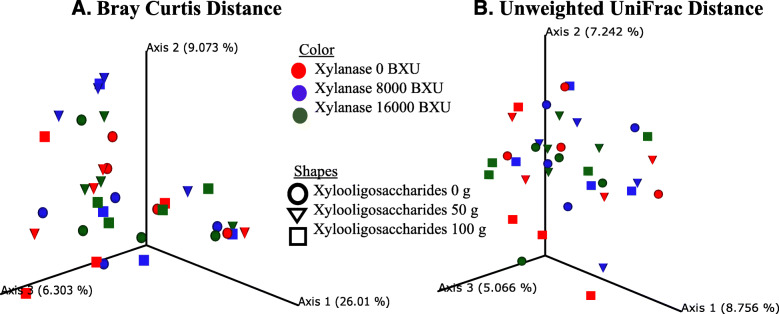
Principal coordinate analysis (PCoA) plot shows beta diversity analysis between different treatments at 10,000 reads depth per sample of cecal contents of broilers from d 42 post-hatch. **a** Bray Curtis distance, **b** Unweighted UniFrac distance. The pairwise comparison did not show any significant differences (*P* > 0.05). Xylanase treatments are represented by colors (red for 0 BXU/kg feed, blue for 8,000 BXU/kg feed, and green for 16,000 BXU/kg feed), and xylooligosaccharides treatments are represented by shapes (circle for 0 g per ton feed, triangle for 50 g per ton feed, and square for 100 g per ton feed)

**Fig. 5:**
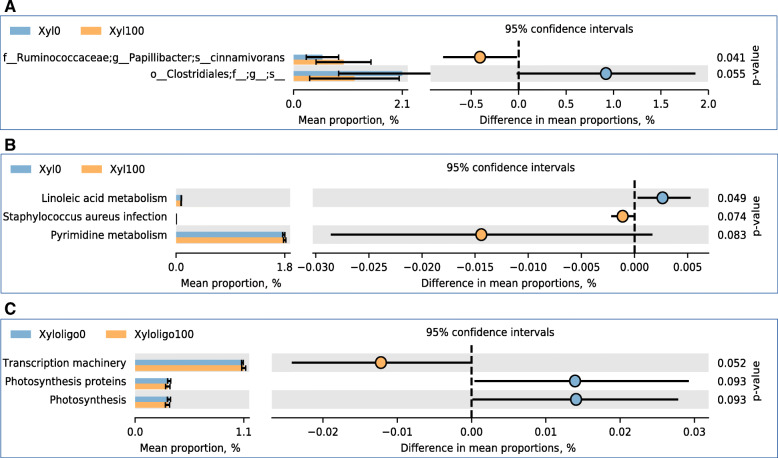
Extended error bar plot showing the mean proportion (%) showing trend or significant variation **a** of different cecal bacteria communities between no xylanase (Xyl0) and 16,000 BXU/kg xylanase (Xyl100) groups in broilers at d 42 post-hatch **b** predicted functions of cecal microbial communities between no xylanase (Xyl0) and 16,000 BXU/kg xylanase (Xyl100) groups in broilers at d 42 post-hatch, and **c** predicted functions of cecal microbial communities between no xylooligosaccharides and 100 g/t xylooligosaccharides groups of broilers at d 42 post-hatch. The sphere points in blue and orange indicate the difference between **a**, **b** no xylanase and 16,000 BXU/kg xylanase groups, and **c** no xylooligosaccharides and 100 g/t xylooligosaccharides groups. The *P*-value on the right was derived from a White’s non-parametric t-test in the statistical analysis of taxonomic and functional profiles (STAMP) software

The immune markers of T-cell (CD3) and B-cell (chB6) in the ileum were not different (*P* > 0.05) across treatments on d 42 (Fig. 1). Compared with control, 100 g/t XOS with no xylanase supplementation expressed a higher level of IL-4 (a T-cell differentiation cytokine) and IL-10 (a marker of anti-inflammatory cytokine) in the ileum (*P* < 0.05). The XOS and xylanase supplementation did not differentially affect the abundance (*P* > 0.05) of any specific phyla of bacteria (Fig. 2). Despite the variability among replicates, Firmicutes was the most abundant phylum, while Bacteroidetes was the second most abundant phylum across all treatments. No significant differences (*P* > 0.05) were observed in pairwise comparison across all treatment combinations when compared for the alpha diversity based on the richness of OTUs, and the richness and evenness of bacterial communities based on the Shannon index (Fig. 3). Likewise, the UniFrac and Bray Curtis measure of beta diversity for the differential communities were not significantly different (*P* > 0.05) between treatments (Fig. 4). However, further microbiota analysis between xylanase 0 BXU/kg and xylanase 16,000 BXU/kg groups revealed a higher (*P* < 0.05) mean proportion of *Papillibacter cinnamivorans* and a trend (*P* = 0.055) for a lower mean proportion of an unclassified bacterial species in the order of *Clostridiales* in the high xylanase group compared with no xylanase group (Fig. 5a).

The predicted functions of cecal microbial communities showed that there was a significant difference (*P* < 0.05) between xylanase 0 BXU/kg and xylanase 16,000 BXU/kg for linoleic acid metabolism (Fig. 5b). Trends (*P* < 0.1) between xylanase 0 BXU/kg and xylanase 16,000 BXU/kg (for *Staphylococcus aureus* infection and pyrimidine metabolism) as well as XOS 0 g/t and XOS 100 g/t (for transcription machinery and photosynthesis proteins) for predicted functions of cecal microbiota was observed (Fig. 5b, c). However, the difference in the mean proportion between these groups for these predicted functions was not large.

**Fig. 6:**
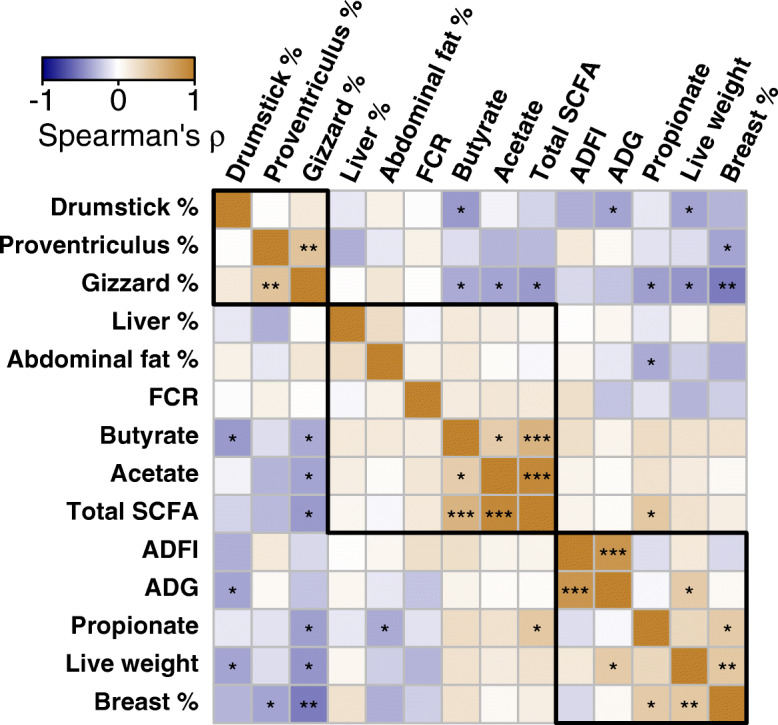
A correlogram showing association among metabolic functions, growth performance parameters, and carcass weight variables across all treatments in broilers at d 42 post-hatch. The spearman’s rho (ρ) correlation coefficient is denoted by the color of the heatmap, and the values are represented in the legend, where the blue color denotes negative correlation while the brown color denotes positive correlation. The asterisk in the cell represents a statistically significant correlation (*P ≤ 0.05, **P ≤ 0.01, ***P ≤ 0.001), and the rectangles with a black border indicate clustering of the parameters. The percentage (%) organ weight denotes the relative organ weight (g/100 g live weight). ADFI: average daily feed intake, ADG: average daily gain, FCR: feed conversion ratio. Live weight = Total body weight at d 42

**Fig. 7:**
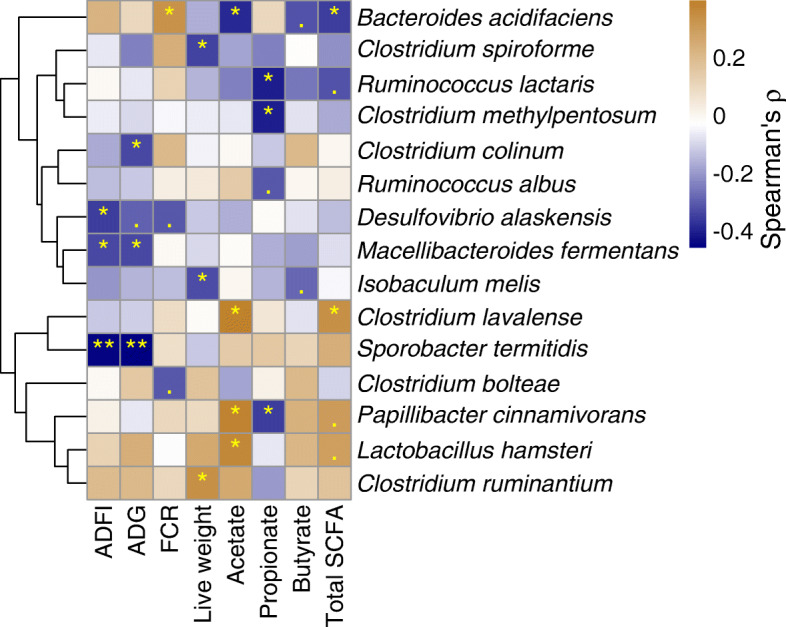
A heatmap showing an association between some cecal bacterial species and biological parameters across all treatments in broilers at d 42 post-hatch. The Spearman’s rho (ρ) correlation coefficient is denoted by the color of the heatmap, and the values are represented in the legend, where the blue color denotes negative correlation while the brown color denotes positive correlation. The yellow asterisks in the cell indicate a statistically significant correlation (*P ≤ 0.05, **P ≤ 0.01), while the dots indicate approaching significance or a trend (*P* < 0.1). The dendrogram is generated by the Euclidean distance method and represents the clustering of bacterial species. ADFI: average daily feed intake, ADG: average daily gain, FCR: feed conversion ratio. Live weight = Total body weight at d 42

The associations between the growth parameters, carcass yield, and metabolites of all treatments displayed a negative correlation of relative weight of drumstick with butyrate, ADG, and live weight (ρ =  − 0.34 to − 0.39; *P* < 0.05; Fig. 6, Table S2). The relative weight of the gizzard was negatively correlated with acetate, propionate, butyrate, and total SCFA, as well as live weight (ρ =  − 0.33 to − 0.41; *P* < 0.05). Abdominal fat was negatively correlated with propionate (ρ =  − 0.33; *P* < 0.05). The relative weight of the breast was positively correlated with propionate and live weight (ρ = 0.41 to 0.43; *P* < 0.05). In correlation analysis of all treatments for the association between cecal microbiota and biological parameters of broilers, FCR was positively correlated with *Bacteroides acidifaciens* (ρ = 0.34; *P* < 0.05; Fig. 7, Table S2). Live weight was correlated positively (ρ = 0.34) with *Clostridium ruminantium,* and negatively (ρ = − 0.33 to − 0.34) with *Clostridium spiroforme* and *Isobaculum melis* (*P* < 0.05). Acetate was correlated positively (ρ = 0.37 to 0.4) with *Papillibacter cinnamivorans*, *Clostridium lavalense,* and *Lactobacillus hasmsteri* and negatively (ρ = − 0.39) with *Bacteroides acidifaciens* (*P* < 0.05). Propionate was correlated negatively (ρ = − 0.35 to − 0.40) with *Papillibacter cinnamivorans*, *Clostridium methylpentosum* and *Ruminococcus lactaris* (*P* < 0.05).

## Discussion

The supplementation of xylanase can improve AME and amino acid digestibility in a wheat-based diet [26], which can subsequently improve the broiler’s growth performance [13]. The exogenous xylanase has also been reported to improve the nutrient digestibility and growth performance in broilers fed corn-based diets [27, 28]. However, the amount of insoluble and soluble NSP present in the digesta and the type of xylanase included in the diet could affect the broilers’ performance [29]. The role of xylanase in improving nutrient utilization in broilers is substantiated by the observed linear improvement in ADG in the finisher and ADG and FBWG in the total period. A similar improvement in the ADG and the final body weight gain by exogenous xylanase was observed in a recent study by Singh et al. [4]. The authors also reported an interaction between additional dietary fiber source and xylanase for FCR, indicating no additional improvement in FCR by xylanase in corn-SBM diet, and this observation is in line with the findings of the present study. It was also observed that 8,000 BXU/kg xylanase generated a comparable improvement in broilers’ performance as 16,000 BXU/kg supplementation. It suggests that this level of xylanase inclusion can degrade the pentosans substrates at a standard fiber content in the broilers feed. Hence it necessitates that the effects of the higher doses of xylanase be evaluated at different increasing levels of dietary fiber as several authors have reported that increasing the level of xylanase in the feed than recommended did not yield further improvements, while in some studies it reduced the growth and nutrients digestibility in broilers [8, 27, 30]. In this study, XOS supplementation produced only a numerical improvement in FBWG and ADG in the total period compared with groups without XOS supplementation. In contrast, XOS has been reported to increase body weight gain in broilers in the study of Zhenping et al. [17]. The differences in the outcomes could be attributed to the variability in the extraction of XOS and a 10 times higher level of XOS used in the study of Zhenping et al. [17] compared with the lower level used in the present study. The supplemental xylanase only had a trend for the increment of VH: CD ratio in the ileum, while XOS did not influence this ratio. The addition of oligosaccharides has also not been found to affect this ratio by Shang et al. [31], while due to the lack of high viscosity in corn-SBM-based diet, the effect of xylanase on this ratio may not have been significantly larger [32].

It is interesting to observe that both XOS and xylanase increased the production of acetate in the ceca, and it indicates their influence in modulating the fermentation characteristics of cecal microbiota. The XOS in this study was supplemented in low concentration that is not expected to produce sufficient SCFA, but the substantial increase in SCFA in response to XOS could be attributed to its priming of cecal microbiota for enhanced fermentation of selective substrates [1]. Recently, a term “stimbiotic” has been proposed to suggest the mechanism by which the oligosaccharides could influence the utilization of similar nutrients and their extraction from the diet by the gut microbiota [33]. It has been interpreted that while these oligosaccharides supplied in the diet or generated in situ in a small quantity are insufficient to contribute to a differentially high amount of SCFA production in the ceca of broilers, but it acts as a signaling molecule that stimulates the microbial adaptation to degrade dietary fiber sources [1]. Moreover, a similar effect of xylanase on the increase of acetate and total SCFA was also reported by Dale et al. [9] and Singh et al. [4]. In a previous study in laying hens, Ding et al. [15] found that XOS has an immunomodulating effect and the increase in IL-4 and IL-10 cytokines in the high XOS group in the present study also supports this observation. However, whether it is a direct effect of XOS or an indirect influence through the metabolites produced by the gut microbiota utilizing XOS substrates remains unexplained. The XOS administration in a mice study has been reported to reduce IL-1β level in the blood, but it did not affect any immune-related genes in the intestine and thus indicates the variability in the immune system's response in different body sites and tissues [34]. The positive correlation of propionate with the relative weight of the breast suggests that this glucogenic SCFA would be an important contributor to breast muscle growth [35, 36]. The negative correlation of relative gizzard weight with the production of SCFA suggests that a reduced grinding force would provide more undigested substrates for bacterial fermentation in the ceca of broilers [37]. The abundance of Firmicutes phylum was above 75% across all treatments, and it is in agreement with that observed by Zhang et al. [38]. In broilers fed corn hulls derived XOS, Samanta et al. [39] reported that XOS reduced the population of *E. coli* and *Streptococci,* and stimulated the increase of *Bifidobacteria.* However, in the present study, such a considerable impact on these microbiotas was not observed in response to either xylanase or XOS. The addition of xylanase increased the *Papillibacter cinnamivorans* bacteria of the family Ruminococcaceae, and the bacteria in this family are known to support the host by producing enzymes to degrade and utilize fiber [40, 41]. The *P. cinnamivorans* can utilize cinnamate, a precursor of lignin, to produce acetate and show a ligninolytic activity [42, 43]. The positive correlation between *P. cinnamivorans* and acetate also supports this underlying mechanism of the utilization of substrates to produce acetate. The positive correlation of acetate production with *Lactobacillus* observed in this study was also reported previously [44], and acetate has been found to be produced from lactate when the glucose resource gets depleted [45]. The positive correlation of *Bacteroides acidifaciens* with FCR and its negative correlation with acetate suggests that the gut microbiome plays roles beyond fermentation and support growth and lipid utilization [46, 47]. In the present study, the scale of the response of xylanase was greater than that of XOS on cecal microbiota and its fermentative production of SCFA. Nonetheless, the lack of significant interaction between xylanase and XOS points to their working by the additive mechanism. However, further studies are warranted to elucidate such variable response of cecal microbiota to the supplementation of xylanase and XOS, and their interaction in broilers.

## Conclusions

The results of this study support the hypothesis that the xylanase supplementation in the corn-SBM-based diet is capable of improving the growth performance in broilers. Moreover, it can be inferred from the results that xylanase and XOS supplementation in the broilers diet can potentially enhance the production of SCFA through increased cecal fermentation. However, such improvements may not necessarily depend on the shift in the microbiota diversity or the enhancement of mucosal absorptive surface. The numerical improvements in the bodyweight by XOS were evident, but it failed to reach statistical significance in the present study, and thus it is recommended that more birds be used in future studies of XOS supplementation in broilers.

## Supplementary Information

**Additional file 1. Table S1.** Spearman correlation between metabolic functions, growth performance parameters, and carcass weight variables across all treatments inbroilers at d 42 post-hatch. **Table S2.** Spearman correlation between the differential bacterial species and biological parameters across all treatments in broilers at d 42 post-hatch.

## Data Availability

Not applicable.
