# Supplementary material for: Effects of supplemental xylanase and xylooligosaccharides on production performance and gut health variables of broiler chickens
Source: J Anim Sci Biotechnol. 2021 Sep 6;12:98. doi: 10.1186/s40104-021-00617-8 (PMC8419990; doi:10.1186/s40104-021-00617-8)
Supplement: Supplementary file 1 — Additional file 1. Table S1. Spearman correlation between metabolic functions, growth performance parameters, and carcass weight variables across all treatments inbroilers at d 42 post-hatch. Table S2. Spearman correlation between the differential bacterial species and biological parameters across all treatments in broilers at d 42 post-hatch. [file 40104_2021_617_MOESM1_ESM.docx]

**Supplementary Information**

**Table S1** Spearman correlation between metabolic functions, growth performance parameters, and carcass weight variables across all treatments in broilers at d 42 post-hatch.

| **Variables** | **By variables** | **Spearman ρ** | **Prob> ρ** |
| --- | --- | --- | --- |
| Acetate | Gizzard: Live weight | -0.345 | 0.039 |
| Propionate | Breast: Live weight | 0.407 | 0.014 |
| Propionate | Gizzard: Live weight | -0.378 | 0.023 |
| Propionate | Abdominal fat: Live weight | -0.334 | 0.047 |
| Butyrate | Acetate | 0.393 | 0.018 |
| Butyrate | Drumstick: Live weight | -0.391 | 0.019 |
| Butyrate | Gizzard: Live weight | -0.335 | 0.046 |
| Total SCFA | Butyrate | 0.586 | 0.000 |
| Total SCFA | Propionate | 0.424 | 0.010 |
| Total SCFA | Gizzard: Live weight | -0.389 | 0.019 |
| Live weight | ADG Total | 0.402 | 0.015 |
| Breast: Live weight | Gizzard: Live weight | -0.502 | 0.002 |
| Breast: Live weight | Live weight | 0.428 | 0.009 |
| Breast: Live weight | Proventriculus: Live weight | -0.358 | 0.032 |
| Drumstick: Live weight | Live weight | -0.353 | 0.035 |
| Drumstick: Live weight | ADG Total | -0.345 | 0.039 |
| Gizzard: Live weight | Proventriculus: Live weight | 0.479 | 0.003 |
| Gizzard: Live weight | Live weight | -0.410 | 0.013 |

*The relative organ weight is expressed as g/100g live weight. ADG: average daily gain, SCFA: short-chain fatty acids. Live weight = Total body weight at d 42.

**Table S2** Spearman correlation between the differential bacterial species and biological parameters across all treatments in broilers at d 42 post-hatch.

| **Variables** | **Differential bacterial species** | **Spearman ρ** | **Prob> ρ** |
| --- | --- | --- | --- |
| ADFI | *Sporobacter termitidis* | -0.442 | 0.007 |
| ADFI | *Desulfovibrio alaskensis* | -0.350 | 0.036 |
| ADFI | *Macellibacteroides fermentans* | -0.331 | 0.049 |
| ADG | *Sporobacter termitidis* | -0.458 | 0.005 |
| ADG | *Macellibacteroides fermentans* | -0.337 | 0.045 |
| ADG | *Clostridium colinum* | -0.333 | 0.047 |
| ADG | *Desulfovibrio alaskensis* | -0.290 | 0.086 |
| FCR | *Bacteroides acidifaciens* | 0.336 | 0.045 |
| FCR | *Clostridium bolteae* | -0.311 | 0.064 |
| FCR | *Desulfovibrio alaskensis* | -0.306 | 0.069 |
| Live weight | *Isobaculum melis* | -0.329 | 0.050 |
| Live weight | *Clostridium ruminantium* | 0.338 | 0.044 |
| Live weight | *Clostridium spiroforme* | -0.342 | 0.041 |
| Acetate | *Clostridium lavalense* | 0.396 | 0.017 |
| Acetate | *Bacteroides acidifaciens* | -0.388 | 0.019 |
| Acetate | *Papillibacter cinnamivorans* | 0.381 | 0.022 |
| Acetate | *Lactobacillus hamsteri* | 0.370 | 0.026 |
| Propionate | *Ruminococcus lactaris* | -0.403 | 0.015 |
| Propionate | *Clostridium methylpentosum* | -0.401 | 0.015 |
| Propionate | *Papillibacter cinnamivorans* | -0.354 | 0.034 |
| Propionate | *Ruminococcus albus* | -0.311 | 0.065 |
| Butyrate | *Bacteroides acidifaciens* | -0.319 | 0.058 |
| Butyrate | *Isobaculum melis* | -0.282 | 0.095 |
| Total SCFA | *Bacteroides acidifaciens* | -0.351 | 0.036 |
| Total SCFA | *Clostridium lavalense* | 0.339 | 0.043 |
| Total SCFA | *Ruminococcus lactaris* | -0.320 | 0.057 |
| Total SCFA | *Papillibacter cinnamivorans* | 0.301 | 0.075 |
| Total SCFA | *Lactobacillus hamsteri* | 0.287 | 0.090 |

*ADFI: average daily feed intake, ADG: average daily gain, FCR: feed conversion ratio, SCFA: short-chain fatty acids. Live weight = Total body weight at d 42.
